# High-intensity physical activity is not associated with better cognition in the elder: evidence from the China Health and Retirement Longitudinal Study

**DOI:** 10.1186/s13195-021-00923-3

**Published:** 2021-11-03

**Authors:** Zhiyuan Wu, Haiping Zhang, Xinlei Miao, Haibin Li, Huiying Pan, Di Zhou, Yue Liu, Zhiwei Li, Jinqi Wang, Xiangtong Liu, Deqiang Zheng, Xia Li, Wei Wang, Xiuhua Guo, Lixin Tao

**Affiliations:** 1 Beijing Municipal Key Laboratory of Clinical Epidemiology, School of Public Health, Capital Medical University, No.10 Xitoutiao, Youanmen Street, Beijing, 100069 China; 2 Centre for Precision Health, School of Medical and Health Sciences, Edith Cowan University, Perth, Australia; 3 Heart Center & Beijing Key Laboratory of Hypertension, Beijing Chaoyang Hospital, Capital Medical University, Beijing, China; 4 Department of Mathematics and Statistics, La Trobe University, Melbourne, Australia

**Keywords:** Cognition, Physical activity intensity, Cohort study, Mixed effect model

## Abstract

**Background:**

To evaluate the association of physical activity (PA) intensity with cognitive performance at baseline and during follow-up.

**Methods:**

A total of 4039 participants aged 45 years or above from the China Health and Retirement Longitudinal Study were enrolled in visit 1 (2011–2012) and followed for cognitive function in visit 2 (2013–2014), visit 3 (2015–2016), and visit 4 (2017–2018). We analyzed the association of PA intensity with global cognition, episodic memory, and mental intactness at baseline using adjusted regression methods and evaluated the long-term effect of PA intensity using multiple measures of cognition scores by mixed effect model.

**Results:**

In cross-sectional analysis, mild and moderate PA, rather than vigorous PA, was associated with better cognitive performance. The results remained consistent in multiple sensitivity analyses. During the follow-up, participant with mild PA had a 0.56 (95% CI 0.12–0.99) higher global cognition, 0.23 (95% CI 0.01–0.46) higher episodic memory, and 0.33 (95% CI 0.01–0.64) higher mental intactness, while those with moderate PA had a 0.74 (95% CI 0.32–1.17) higher global score, 0.32 (95% CI 0.09–0.54) higher episodic memory, and 0.43 (95% CI 0.12–0.74) higher mental intactness, compared with individuals without PA. Vigorous PA was not beneficial to the long-term cognitive performance.

**Conclusions:**

Our study indicates that mild and moderate PA could improve cognitive performance, rather than the vigorous activity. The targeted intensity of PA might be more effective to achieve the greatest cognition improvement considering age and depressive status.

**Supplementary Information:**

The online version contains supplementary material available at 10.1186/s13195-021-00923-3.

## Background

Decline in cognitive function is one of the main causes of disability and even death in the elderly, which has a pernicious impact on physical and mental health, independent living ability and social function. In China, the prevalence rates of mild cognitive impairment (MCI) and dementia are rapidly rising along with the population aging, and approximately 5.14% of people over 65 years had dementia [1]. Dementia has caused a heavy socio-economic burden, with the global cost of dementia is reported to be US $957.56 billion in 2015, set to reach US $2.54 trillion by 2030 and US $9.12 trillion by 2050 [2]. Aging is a major risk factor of cognitive function decline and cognitive impairment progression, and evidence suggests that some important cognitive functions such as episodic memory decline with age even in the middle-aged people [3]. Thus, strengthening the prevention of cognitive decline and proposing the valid strategy to avoid cognitive impairment is of great public health significance.

Physical activity (PA) is associated with various health benefits, including cardiopulmonary capacity and muscular endurance, and the reduced risk of overall mortality, cardiovascular diseases, metabolic syndrome, and psychiatric disorders [4–6]. As a modifiable factor, PA acts as a non-pharmacological strategy to mitigate the age-related impairment on cognitive performance [7]. However, there exists a large amount of variation in its effect on cognition improvement [8, 9]. The effects of PA on improving cognitive function have been extensively studied in different populations [7]. In a meta-analysis of 39 randomized controlled trials (RCT), the results showed that PA interventions are effective for improving cognitive function in older adults regardless of the baseline cognitive status [10]. In addition, a RCT in older adults aged 55 to 80 years without psychiatric or neurological diseases showed a positive association of cognitive improvement with aerobic fitness and training-induced neuroplasticity. However, in another RCT of exercise interventions, the researchers did not find sufficient evidence that PA or aerobic fitness could improve the cognitive performance in older adults over 50 years [11]. The dose of PA volume, duration, frequency, or intensity, together with the population features, are possible factors contributing to this variation [9], and the dose-response relationship between PA and the positive outcome was always argued [10, 12, 13]. The 2018 Physical Activity Guidelines stated that little is known about the dose of PA intensity needed to improve cognitive function yet, and there lacks strong evidence that moderate-to-vigorous PA could improve the cognition, especially in the middle-aged adults [7]. Sammi et al. found that more exercise is not always better for the self-reported mental health burden [6]. However, it remains unknown whether or not high-intensity PA is related to better cognitive performance [14, 15]. The long-term effect of PA on cognitive performance remains unclear neither.

In this study, we explored the association of PA intensity with global cognition, episodic memory and mental intactness both at baseline and during follow-up, after accounting for a range of sociodemographic characteristics and physical health conditions.

## Methods

### Data sources and study design

This current study was a secondary analysis of the China Health and Retirement Longitudinal Study (CHARLS), which is a national prospective cohort collecting a wide range of social and economic data, personal health information for geriatric and health policy research (http://charls.pku.edu.cn/). A total of 17,705 participants from 150 counties or districts within 28 provinces in China were recruited in the demographic background survey at baseline (visit 1: 2011–2012) and followed up every 2 years at visit 2 (2013–2014), visit 3 (2015–2016), and visit 4 (2017–2018). Details of the cohort design have been described previously [16]. The CHARLS study was approved by the institutional review board of Peking University (IRB00001052-11015). Written informed consent was obtained before participation. At each visit, the trained staff conducted face-to-face interviews to collect the sociodemographic characteristics, medical history, health behavior, cognitive function, and depressive status using standardized questionnaire.

**Fig. 1:**
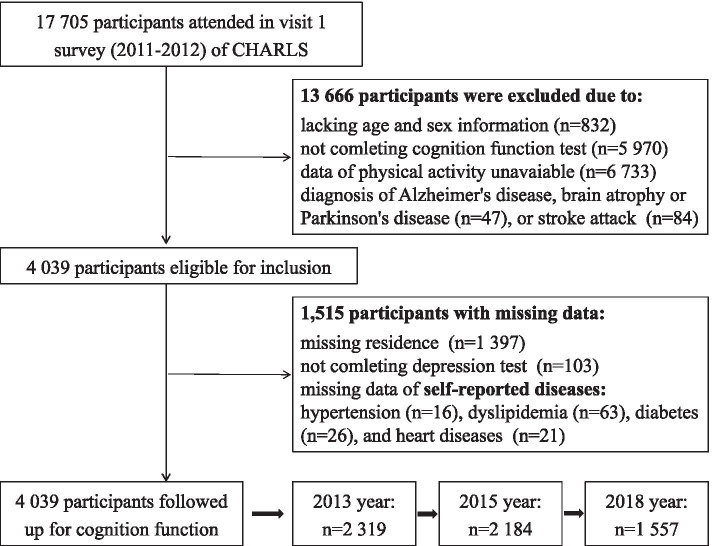
Flow chart of this current study

Of 17,705 participants at baseline, we excluded 832 individuals without age and sex information, or younger than 45 years. Then, 5970 and 6733 participants lacking cognitive function data or PA intensity information were excluded, as the physical activity investigation was limited to a randomly selected subgroup in the CHARLS study. We excluded 47 individuals with Alzheimer's disease, brain atrophy, or Parkinson’s disease, and 84 individuals with history of stroke. Finally, 4039 participants were included for this current analysis, and 2319, 2184, and 1557 of them provided cognitive function data during follow-up at visit 2, visit 3, and visit 4, respectively. The flow chart of this study was shown in Fig. 1.

### Cognition measurement

In accordance with previous studies [17–19], the cognitive performance was tested by two cognition measures in this study: episodic memory and mental intactness. The episodic memory reflects an individual’s ability to immediately repeat ten Chinese words just read to them in any order (referred as immediate recall) and to recall the same words four minutes later (referred as delayed recall). The episodic memory score is the mean value of immediate and delayed recall scores, and ranges from 0 to 10. The mental intactness reflects the mental status based on several questions of the Telephone Interview of Cognitive Status (TICS) battery, including serial subtraction of 7 from 100 (up to five times), the date (month, day, year and season), the day of week, and the ability to redraw a picture shown to the individual. Answers to these questions are summed into the mental intactness score ranging from 0 to 11. The global cognition score is calculated as the sum of episodic memory score and mental intactness score, which ranges from 0 to 21.

### Definition of PA intensity

PA was quantified via a modified short form of the physical activity questionnaire [20]. Participants were asked to report the number of days and typical spent time-per-day for three activity types (mild, moderate and vigorous) during the usual week. Having “mild,” “moderate,” or “vigorous” PA was defined as having the corresponding activity type on three or more days per week and for at least 10 min at every time. Participants were classified into “none,” “mild,” “moderate,” and “vigorous” PA intensity according to the highest intensity they reported. The vigorous activities make breathe much harder than normal and include heavy lifting, digging, plowing, aerobics, fast bicycling, and cycling with a heavy load; the moderate activities make you breathe somewhat harder than normal and include carrying light loads, bicycling at a regular pace, mopping the floor, doing Taiji, and walking fast; spend walking in a usual week; the mild activity refers to waking at work, at home, walking to travel from place to place, and any other walking that you might do solely for recreation, sport, exercise, or leisure. Participants without any of these activity types or not meeting the relevant standards were classified as “none” PA intensity.

### Covariates

Baseline measurements of age, sex, education level, marital status, residence location, BMI, smoking, drinking, self-reported health conditions and medication use, and depression status were included as covariates in the current study. Educational level was categorized as “primary education,” “secondary education,” and “third education.” Marital status included “married” and “others.” Residence location included “urban” and “rural.” Smoking status was defined as “never smoking,” “current smoker,” and “former smoker.” Drinking status was defined as “current drinking more than once per month,” “current drinking once or less than once per month,” and “no current drinking.” Self-reported health conditions included the diagnosis of hypertension, diabetes, dyslipidemia, and heart diseases (heart attack, coronary heart disease, angina, congestive heart failure, or other heart problems). Depressive symptoms were assessed using the 10-item version of the Epidemiologic Studies Depression Scale (CES-D), and a score of ≥ 12 indicated the presence of depressive symptoms [21]. BMI was calculated as weight (in kilograms)/height^2 (in meter squared), and grouped into underweight (BMI < 18.5 kg/m^2^), normal weight (BMI 18.5–23.9 kg/m^2^), overweight (BMI ≥ 24.0 kg/m^2^) according to the overweight and obesity standard [22].

### Statistics analysis

Baseline characteristics are presented as the mean (standard deviation, SD), median [interquartile range, IQR] or number (percentage), as appropriate. To show the distribution differences, we compared the scores of global cognition, episodic memory, and mental intactness among those with different PA intensity by Kruskal-Wallis test, both with the population without PA and the whole population as the reference.

To investigate the association of PA intensity and cognitive performance at baseline, we performed adjusted analyses using the regression models for the individual-level factors: model 1 was adjusted for age; model 2 was adjusted for age, sex, BMI, education level, marital status, residence location, health conditions, smoking, current drinking, and CES-D score. To validate the findings, we did multiple sensitivity analyses. First, 37.5% (1515 of 4039) of total data items were missing at residence type (*n* of missing 1397), health conditions (*n* of missing 16 for hypertension, 63 for dyslipidemia, 26 for diabetes, 21 for heart diseases), or CES-D assessment (*n* of missing: 103). Thus, we repeated the analyses using the imputed data by multiple imputation of chained equations method. We created five imputed data sets and pooled the results. Second, to account for the imbalanced self-select probability into different PA intensity group, we applied a multigroup propensity score weighting procedure using the tree-based regression model. The covariate set of age, sex, BMI, education, marital status, residence, and CES-D score, which were significantly associated with cognitive performance in the analyses, were considered in this weighting procedure. The max number of trees was set as 10000 iterations. The balance measures of interest corresponding with iterations were shown in Fig. S1 to ensure that the parameter was reasonable.

To investigate the long-term effect of PA intensity on cognitive performance during follow-up, we analyzed the association of PA intensity at baseline with multiple measures of cognition scores using mixed effect model. The main effect of PA intensity and visit time, together with the interaction term, were fitted in the mixed model. Mixed effect model offers a better way to deal with missing data, and subjects with more missing values have a weaker impact on parameter estimation. The same covariates were adjusted as in model 2, and the individual difference was considered as a random effect term in the analyses. Then, we analyzed the effect of PA intensity in certain subgroups, according to age (< 60 years and ≥ 60 years), sex (male and female), smoking, drinking, BMI level, and depression status (CES-D < 12 and CES-D ≥ 12). All the analyses presented above were conducted using packages of “mice,” “twang,” “survey,” and “lmerTest” by R software (version 4.1.0).

### Data availability

The CHARLS dataset is freely available to all researchers in related fields on request. Researchers can gain access to the data (http://charls.pku.edu.cn/). And the datasets used and/or analyzed in this current study are available from the corresponding author (Dr. Lixin Tao) on reasonable request.

## Results

### Participant characteristics

**Fig. 2:**
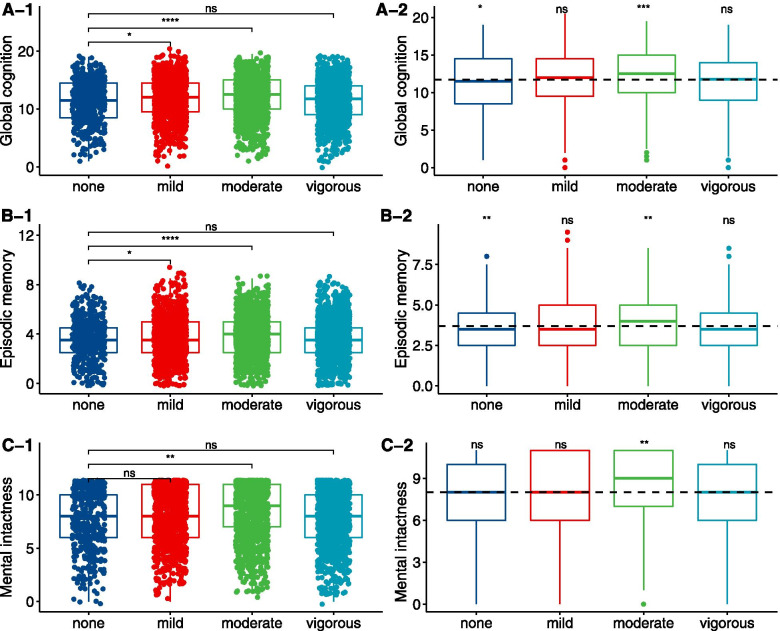
Distribution of cognition scores among participants according to physical activity intensity. The distributions were compared with the group of none physical activity as reference (A-1, B-1, C-1), with the whole population as reference (A-2, B-2, C-2)

The demographic information of the included participants and excluded participants is shown in Table S1, and the distribution of age and sex is similar. This current study was based on 4039 participants, and 2050 (50.8%) were male. The mean age was 58.12 (SD 9.12) years. The population level of global cognition, episodic memory, and mental intactness were 11.71 (SD 3.57), 3.71 (SD 1.67), and 8.01 (SD 2.64). Vigorous PA was reported by 1 306 (32.3%) participants, while 1263 (31.3%), 1002 (24.8%), and 468 (11.6%) reported moderate, mild, and none PA, respectively. Full demographic characteristics and health conditions of the study population are presented in Table 1. Individuals with vigorous PA were younger, being male, from a rural residence, having a lower education, current smoking, and drinking as shown in Table S2. Individuals with moderate PA showed higher global cognition, episodic memory, and mental intactness scores, both compared with those of none PA and the whole population. Noticeably, there were no significant differences regarding cognitive performance in individuals of vigorous PA as shown in Fig. 2.

**Table 1:** Baseline characteristics of the study population

	Overall (*n* = 4039)	Male (*n* = 2050)	Female (*n* = 1989)	*P* value
**Cognitive function**				
Episodic memory	3.71 (1.67)	3.72 (1.62)	3.69 (1.73)	0.605
Mental intactness	8.01 (2.64)	8.46 (2.41)	7.54 (2.78)	< 0.001
Global cognition	11.71 (3.57)	12.18 (3.30)	11.23 (3.76)	< 0.001
**Physical activity**				< 0.001
None	468 (11.6)	211 (10.3)	257 (12.9)	
Mild	1002 (24.8)	471 (23.0)	531 (26.7)	
Moderate	1263 (31.3)	577 (28.1)	686 (34.5)	
Vigorous	1306 (32.3)	791 (38.6)	515 (25.9)	
**Age (years)**	58.12 (9.12)	59.26 (9.01)	56.95 (9.10)	< 0.001
**Education level**				
Primary	2536 (62.8)	1128 (55.0)	1408 (70.8)	< 0.001
Secondary	1421 (35.2)	865 (42.2)	556 (28.0)	
Third	82 (2.0)	57 (2.8)	25 (1.3)	
**Marital status**				< 0.001
Married	3613 (89.5)	1874 (91.4)	1739 (87.4)	
Other	426 (10.5)	176 (8.6)	250 (12.6)	
**Residence**				< 0.001
Urban	589 (22.3)	251 (17.5)	338 (28.0)	
Rural	2053 (77.7)	1186 (82.5)	867 (72.0)	
**BMI (kg/m**^**2**^**)**	23.49 (4.00)	23.39 (3.92)	23.59 (4.07)	0.101
**Health conditions**				
Hypertension	929 (23.1)	493 (24.9)	436 (21.4)	0.009
Diabetes	224 (5.6)	92 (4.5)	132 (6.7)	0.004
Dyslipidemia	366 (9.2)	170 (8.4)	196 (10.0)	0.096
Heart diseases	447 (11.1)	184 (9.0)	263 (13.3)	< 0.001
**Smoking status**				< 0.001
Current	1298 (32.1)	1180 (57.6)	118 (5.9)	
Former	365 (9.0)	327 (16.0)	38 (1.9)	
Never	2376 (58.8)	543 (26.5)	1833 (92.2)	
**Current drinking**				< 0.001
> Once a month	1115 (27.6)	961 (46.9)	154 (7.7)	
≤ Once a month	335 (8.3)	213 (10.4)	122 (6.1)	
No current drinking	2589 (64.1)	876 (42.7)	1713 (86.1)	
**CES-D score**	6.00 [3.00, 11.00]	6.00 [3.00, 10.00]	7.00 [4.00, 12.00]	< 0.001

Data are the mean (SD), median [IQR] or number (%), as appropriate

### Association between PA intensity and cognition at baseline

Based on the adjusted regression model, compared with individuals without PA, those with mild PA had 0.42 (95% CI 0.07–0.77) higher global cognition (3.7% increment) and 0.20 (95% CI 0.03–0.38) higher episodic memory (5.9% increment), while individuals with moderate PA had 0.59 (95% CI 0.26–0.93) higher global cognition (5.3% increment), 0.25 (95% CI 0.08–0.41) higher episodic memory (7.1% increment), and 0.34 (95% CI 0.09–0.60) higher mental intactness (4.4% increment) at baseline. Vigorous PA was not associated with better global cognition, neither its components (all *P* > 0.05) as shown in Table 2. In the multivariate model, age and CES-D score had negative effects on the cognitive performance, while those of male, higher education, married status, living in urban area, and nondrinker had better cognitive performance (Table S3).

**Table 2:** Association of physical activity intensity with global cognition, episodic memory, and mental intactness at baseline

	Model 1			Model 2		
	β	95% CI	*P* value	β	95% CI	*P* value
**Global cognition**						
Physical activity (ref: none)						
Mild	0.575	0.197–0.953	**0.003**	0.421	0.073–0.768	0.018
Moderate	0.631	0.265–0.998	**< 0.001**	0.59	0.255–0.926	< 0.001
Vigorous	− 0.124	− 0.490–0.242	0.506	0.133	− 0.209–0.474	0.447
**Episodic memory**						
Physical activity (ref: none)						
Mild	0.268	0.091–0.445	**0.003**	0.204	0.033–0.375	**0.020**
Moderate	0.259	0.088–0.431	**0.003**	0.248	0.082–0.413	**0.003**
Vigorous	− 0.018	− 0.190–0.153	0.835	0.128	− 0.040–0.297	0.135
**Mental intactness**						
Physical activity (ref: none)						
Mild	0.307	0.023–0.592	**0.034**	0.217	− 0.050–0.483	0.111
Moderate	0.372	0.096–0.648	**0.008**	0.343	0.085–0.601	**0.009**
Vigorous	− 0.106	− 0.381–0.169	0.451	0.004	− 0.258–0.266	0.975

Model 1: adjusted for age (*n* = 4 039)

Model 2: adjusted for age, sex, BMI, education level, marital status, residence location, health conditions, smoking, current drinking, and CES-D score (*n* = 2 524)

The results did not significantly change using the imputed data set. Compared with individuals without PA, those with mild PA had a 0.44 higher global cognition score (before imputation 0.42) and 0.22 higher episodic memory (before imputation 0.20), while individuals with moderate PA had a 0.63 higher global cognition score (before imputation 0.59), 0.26 higher episodic memory (before imputation 0.25), and 0.37 higher mental intactness (before imputation 0.34). Vigorous PA was still not associated with global cognition and its components (all *P* > 0.05) as shown in Fig. S2 (A). In the propensity score weighted population, the effective sample size was 3482, and the vigorous PA was still not significantly associated with global cognition, episodic memory, and mental intactness as shown Fig. S2 (B, C).

### Long-term effect of PA intensity on cognition function

During the follow-up, 2319, 2184, and 1557 participants provided cognitive performance data at visit 2, visit 3, and visit 4, as shown in Fig. S3. Compared with individuals without PA, those with mild PA had a 0.56 (95% CI 0.12–0.99) higher global cognition score, 0.23 (95% CI 0.01–0.46) higher episodic memory score, and 0.33 (95% CI 0.01–0.64) higher mental intactness score considering multiple measurements and the long-term effect. Individuals with moderate PA had a 0.74 (95% CI 0.32–1.17) higher global cognition score, 0.32 (95% CI 0.09–0.54) higher episodic memory score, and 0.43 (95% CI 0.12–0.74) higher mental intactness score. Vigorous PA was not associated with long-term improved global cognition and its components (all *P* > 0.05) as shown in Table 3.

**Table 3:** The effect of physical activity intensity on the long-term cognitive performance during follow-up

	Model 1			Model 2		
	*β*	95% CI	*P* value	*β*	95% CI	*P* value
**Global cognition**						
Physical activity (ref: none)						
Mild	0.545	0.178–0.912	**0.004**	0.556	0.119–0.993	**0.013**
Moderate	0.869	0.514–1.224	**< 0.001**	0.744	0.317–1.171	**0.001**
Vigorous	0.200	− 0.153–0.553	0.267	0.24	− 0.187–0.667	0.272
**Episodic memory**						
Physical activity (ref: none)						
Mild	0.253	0.065–0.441	**0.008**	0.23	0.005–0.457	**0.048**
Moderate	0.376	0.194–0.558	**< 0.001**	0.316	0.093–0.539	**0.005**
Vigorous	0.141	− 0.039–0.321	0.128	0.11	− 0.113–0.333	0.336
**Mental intactness**						
Physical activity (ref: none)						
Mild	0.292	0.029–0.555	**0.029**	0.325	0.007–0.643	**0.045**
Moderate	0.493	0.238–0.748	**< 0.001**	0.429	0.117–0.741	**0.007**
Vigorous	0.059	− 0.194–0.312	0.646	0.13	− 0.182–0.442	0.413

Model 1: the main effect of PA intensity and visit time plus the interaction were fitted (*n* = 4 039)

Model 2: model 1 and adjusted for age, sex, BMI, education level, marital status, residence location, health conditions, smoking, current drinking, and CES-D score (*n* = 2 524)

**Fig. 3:**
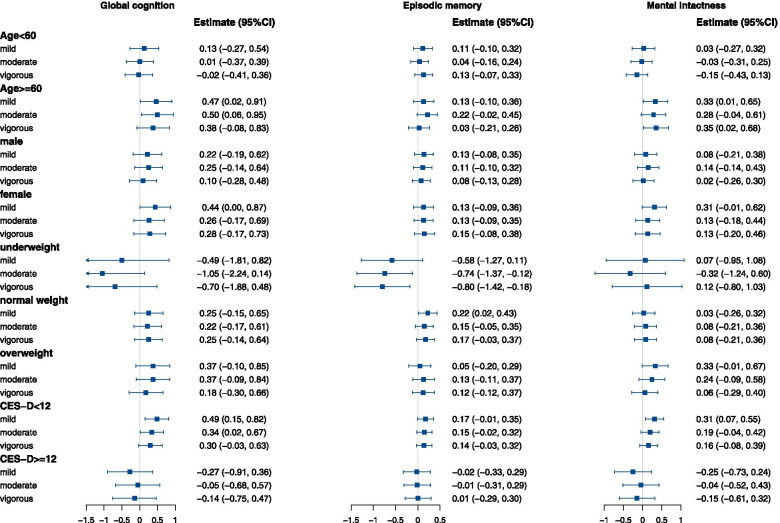
Longitudinal association of physical activity intensity with cognitive performance in subgroups. Adjusted for age, sex, BMI, education level, marital status, residence location, health conditions, smoking, drinking, and CES-D score if not stratified

In the subgroup analyses (Fig. 3), we found the associations varied in certain populations, and the effect of PA intensity was significantly modified by age and depression status in terms of the cognitive performance (*P* for interaction < 0.05). The protective effects of mild and moderate PA were significant dominantly in the elder adults (≥ 60 years) and non-depressive populations. In the elder adults, mild and moderate PA were associated with 0.47 (95% CI 0.02–0.91) and 0.50 (95% CI 0.06–0.95) higher global cognition scores compared to individuals with none PA. Among non-depressive population, global cognition was 0.49 (95% CI 0.15–0.82) higher for those with mild PA, and 0.34 (95% CI 0.02–0.67) higher for those with moderate PA, compared with those without PA.

In addition, we analyzed the association of the frequency of vigorous, moderate, and mild PA with cognition performance separately, and we found that 3–5 days/week for vigorous, moderate, and mild PA was related with higher global cognitive score, while the effect of 1–2 days/week and 6–7 days/week PA was similar to none activity as shown Table S4.

## Discussion

In this cohort study, we found that the mild and moderate intensity of PA are associated with better cognitive performance, rather than vigorous. The results were almost consistent in multiple sensitivity analyses. In addition, the long-term effects of mild and moderate PA on cognitive performance are significant, instead of vigorous activity. The associations of mild and moderate PA are predominant in the elder (≥ 60 years) and non-depressive populations.

To date, numerous studies have investigated the relationship between PA and cognitive function, but the results are contradictory [7, 23]. Results of a meta-analysis of 36 RCTs showed that only moderate (0.17, 95% CI 0.03–0.33) to vigorous (0.16, 95% CI 0.04–0.27) activity improved cognitive function in older adults [10]. While another meta-analysis of prospective studies showed that low to moderate intensity was also associated with a significantly lower risk of cognitive decline compared with inactivity individuals [23]. In our study, we observed only the mild to moderate intensity was associated with better baseline and long-term cognitive performance, which was consistent with several RCTs and meta-analyses that have demonstrated an inverted U-shaped dose-response relationship between PA intensity and cognitive performance [14]. This relationship was first proposed by Yerkers et al.’s arousal performance theory [24]. In recent years, Dietrich et al. proposed that moderate PA could activate the arousal mechanism of the reticulum system, thus improving several domain-specific cognition [25]. However, vigorous PA requires greater activation of the premotor cortex and supplementary motor areas at the expense of the prefrontal cortex, resulting in the disengagement of higher-order functions in the prefrontal cortex. This model provides a neurobiological basis for the dose-response relationship. In addition, the CHARLS study included a higher proportion (77.7% in our study) of rural participants, and the distribution of PA intensity was unbalanced. People from the rural areas were more likely to have higher intensity activity in their daily farming work (heavy lifting, digging, plowing), and the rural participants were vulnerable to lower cognitive score due to limited education and income. Nonetheless, the classification of PA intensity, cognitive testing scale, or the reporting bias could also contribute to the insignificant relationship of vigorous PA and cognition. In other research areas such as mental health, studies have also found that more activity does not necessarily translate into better mental well-being [6].

The underlying mechanisms by which PA promotes the improvement of cognitive function have not been determined yet [9]. One of the key hypotheses is that activity of the sympathetic adrenal system induced by PA increases plasma catecholamine (E) and norepinephrine (NE) concentrations, which feed back to the hypothalamus via the autonomic nervous system, leading to an increase in the catecholamine neurotransmitters (NE) and dopamine (DA) in the brain [26, 27]. The norepinephrine system activated by NE and the dopaminergic pathway activated by DA are both important neurotransmitters related to cognitive function in humans [28]. Furthermore, studies suggest that brain-derived neurotrophic factor (BDNF) also plays an important role in the improvement of cognitive function caused by PA [29]. BDNF can promote neuronal differentiation and survival, which are important moderators of synaptic plasticity [29–31]. PA can increase the expression of BDNF genes and proteins in the cerebellum, cerebral cortex, and hippocampus [32, 33] and induces hippocampal angiogenesis by promoting insulin-like growth factor (IGF-1) and vascular endothelial growth factor (VEGF) production in the periphery [34, 35]. Intervention trials in the elderly have shown that these neurotrophins contribute to the positive effects of PA on cognition [36, 37]. In the existing RCTs of cognitive change caused by PA, the duration of follow-up visit is generally less than 1 year [13, 15]. Compared with measuring the immediate response of cognition to a single PA experience or the effect of PA on cognition over a short period (weeks or months), long-term follow-up visit is more likely to reflect real changes in the individual's cognitive level and the cumulative effect of PA on cognitive function. In this current study, we reported the long-term effect of mild and moderate activity on cognition and validated the observed results.

The stratified analysis results showed that there was a dominantly significant association of mild and moderate activity with cognitive performance among people aged 60 and older, rather than people younger than 60. Most systematic review or meta-analysis of the association between PA and cognitive performance in older adults have yielded positive results [7] and numerous studies have identified the neurobiological mechanisms of cognitive decline in the elder [12, 13, 38]. Strong evidence for biomarker analysis of DA indicates that the density of postsynaptic D1 and D2 receptors in the substantia nigra striatum DA system decreases with age [39], while PA is positively associated with dopamine receptor availability [40–42]. This may account for the greater benefits of PA on cognitive function in the elderly with aging-related impaired DA systems. The results also indicate a gender difference in response to PA intensity. Correspondingly, a meta-analysis of RCTs suggests that the greater benefit of PA in enhancing cognitive function is observed in female, as evidenced by the higher cognitive performance benefit of PA in studies with a higher proportion of female [8]. For the overweight people, only moderate PA showed a positive association with cognitive performance. And in the stratified analysis of depressive status, no significant association between PA and cognitive performance was observed in individuals with depressive symptoms: the neuroplasticity damage caused by depression as well as neuronal atrophy and synaptic loss in the hippocampus leads to poor cognitive performance [43]. In summary, results showed that maintaining a good physical and mental status is significant for the cognitive performance.

The strengths of this study include that we used repeated and well-validated measures of cognitive performance from a national longitudinal cohort. We applied the adjusted regression method and mixed model to analyze the association of PA intensity with cognitive performance at baseline and during follow-up. In addition, we used the multinomial propensity score weighting procedure to account for a range of confounding factors.

### Limitations

We acknowledge several limitations of this study. First, this study is an observational study, and the causality or the intervention effect could not be referred, although we used a series of methods to mitigate the confounding bias. Our findings need further validation in other population-based cohort or intervention research. Second, although the frequency and duration time of vigorous, moderate, and mild PA were measured using a standard questionnaire, the self-reported PA intensity was not an objective measure of activity, and we failed to calculate the individual-level metabolic equivalent value (MET) due to lacking the detailed data of continuous duration time. Further work using more objective PA measures like wearable monitoring devices would hopefully validate the observed association in this study. Third, the assessment tool for cognition ability used at the baseline survey of CHARLS was relatively simple and limited. We adopted 20 items of the Telephone Interview Cognitive Status scale (TICS-40) and one item of the graph drawing test to evaluate the cognition ability of participants. Nevertheless, the neurocognitive testing tools, such as the TICS, Community Screening Instrument for Dementia (CSID), and Mini-Mental State Exam (MMSE), have been used from the post-2017 CHARLS survey, which allows to obtain a more comprehensive assessment of cognitive function.

## Conclusions

In brief, our findings indicate that mild and moderate PA are associated with better cognitive performance, rather than vigorous activity. The significant association is still observed during the long-term follow-up visit of cognitive function. The targeted intensity of PA might be more effective to achieve the greatest cognition improvement in certain population.

## Supplementary Information

**Additional file 1: Table S1.** Baseline characteristics of participants included and excluded in the study. **Table S2.** Baseline characteristics of participants according to the PA intensity. **Table S3.** Full regression results in terms of global cognition, episodic memory and mental intactness. **Table S4.** Association of volume of physical activity intensity with cognition scores. **Figure S1.** The balance measures of interest at different interactions. **Figure S2.** Results of sensitivity. **Figure S3.** The missing pattern of cognition function tests during follow-up.

## Data Availability

The data that support the findings of this study are available in China Health and Retirement Longitudinal Study (CHARLS), at http://charls.pku.edu.cn/. Materials are available on request to the corresponding author.
