# Supplementary material for: High-intensity physical activity is not associated with better cognition in the elder: evidence from the China Health and Retirement Longitudinal Study
Source: Alzheimers Res Ther. 2021 Nov 3;13:182. doi: 10.1186/s13195-021-00923-3 (PMC8567563; doi:10.1186/s13195-021-00923-3)
Supplement: Supplementary file 1 — Additional file 1: Table S1. Baseline characteristics of participants included and excluded in the study. Table S2. Baseline characteristics of participants according to the PA intensity. Table S3. Full regression results in terms of global cognition, episodic memory and mental intactness. Table S4. Association of volume of physical activity intensity with cognition scores. Figure S1. The balance measures of interest at different interactions. Figure S2. Results of sensitivity. Figure S3. The missing pattern of cognition function tests during follow-up. [file 13195_2021_923_MOESM1_ESM.pdf]

Supplementary materials

Table S1: Baseline characteristics of participants included and excluded in the study.

	Participants included (n=4309)	Participants excluded (n=13666)
Age (years)	58.12(9.12)	59.34(10.41)
Sex (n, %)		
missing	0(0.0)	16(0.1)
Female	1989(49.2)	7229(52.9)
Male	2050(50.8)	6421(47.0)
area (n, %)		
missing	1397(34.6)	6086(44.5)
rural	2053(50.8)	5662(41.4)
urban	589(14.6)	1918(14.0)
Education (n, %)		
missing	0(0.0)	55(0.4)
Primary	2536(62.8)	9217(67.4)
Secondary	1421(35.2)	4047(29.6)
Third	82(2.0)	347(2.5)
Marriage (n, %)		
missing	0(0.0)	33(0.2)
married	3613(89.5)	11884(87.0)
other	426(10.5)	1749(12.8)
BMI (kg/m2)	23.49(4.00)	23.47(3.95)
smoking status (n, %)		
missing	0(0.0)	151(1.1)
current	1298(32.1)	4225(30.9)
former	365(9.0)	1075(7.9)
never	2376(58.8)	8215(60.1)
drinking status (n, %)		
missing	0(0.0)	158(1.2)
≤once a month	335(8.3)	1049(7.7)
>once a month	1115(27.6)	3268(23.9)
no current drinking	2589(64.1)	9191(67.3)
Health conditions (n, %)		
Hypertension	918(22.7)	3366(24.6)
Diabetes	223(5.5)	770(5.6)
Dyslipidemia	366(9.1)	1229(9.0)
heart diseases	447(11.1)	1646(12.0)
CES-D score	6.00[3.00,11.00]	7.00[3.00,13.00]

Data are the mean (SD), median [IQR] or number (%), as appropriate.

Table S2: Baseline characteristics of participants according to the PA intensity.

	none (n=468)	mild (n=1002)	moderate (n=1263)	vigorous (n=1306)	P value
Age (years)	59.89(10.07)	60.20(9.68)	57.44(8.97)	56.55(8.03)	<0.001
Sex, Male (n,%)	211(45.1)	471(47.0)	577(45.7)	791(60.6)	<0.001
Residence (n, %)					
urban	82(27.2)	246(36.1)	205(25.8)	56(6.5)	<0.001
rural	219(72.8)	435(63.9)	591(74.2)	808(93.5)	
Education level (n, %)					
primary	284(60.7)	622(62.1)	742(58.7)	888(68.0)	<0.001
secondary	178(38.0)	345(34.4)	487(38.6)	411(31.5)	
third	6(1.3)	35(3.5)	34(2.7)	7(0.5)	
Married (n, %)	402(85.9)	844(84.2)	1144(90.6)	1223(93.6)	<0.001
BMI (kg/m2)	23.63(4.38)	23.70(3.86)	23.48(4.22)	23.29(3.72)	0.085
smoking (n, %)					
current	135(28.8)	284(28.3)	348(27.6)	531(40.7)	<0.001
former	40(8.5)	106(10.6)	114(9.0)	105(8.0)	
never	293(62.6)	612(61.1)	801(63.4)	670(51.3)	
drinking (n, %)					
>once a month	110(23.5)	220(22.0)	330(26.1)	455(34.8)	<0.001
≤once a month	31(6.6)	75(7.5)	113(8.9)	116(8.9)	
no current drinking	327(69.9)	707(70.6)	820(64.9)	735(56.3)	
Hypertension (n, %)	133(28.6)	298(29.9)	283(22.4)	215(16.6)	<0.001
Diabetes (n, %)	27(5.8)	84(8.4)	71(5.6)	42(3.3)	<0.001
Dyslipidemia (n, %)	48(10.4)	128(12.9)	129(10.4)	61(4.8)	<0.001
Heart (n, %)	61(13.1)	162(16.2)	152(12.1)	72(5.6)	<0.001
CES-D score	7.00 [3.00,12.00]	6.00 [3.00,11.00]	6.00 [3.00,11.00]	7.00 [3.00,12.00]	0.06

Data are the mean (SD), median [IQR] or number (%), as appropriate.

Table S3: Full regression results in terms of global cognition, episodic memory and mental intactness**.**

	Global cognition		Episodic memory		Mental intactness	
	β	P value	β	P value	β	P value
Age	-0.0648	**<0.0001**	-0.0325	**<0.0001**	-0.0323	**<0.0001**
BMI	0.0040	0.7470	0.0002	0.9706	0.0038	0.6918
physical activity (ref: none)						
mild	0.4208	0.0175	0.2040	0.0195	0.2167	0.1110
moderate	0.5906	0.0006	0.2476	0.0034	0.3430	0.0091
vigorous	0.1325	0.4468	0.1283	0.1353	0.0041	0.9754
urban (ref: rural)	0.8153	**<0.0001**	0.2736	**0.0007**	0.5418	**<0.0001**
Male (ref: female)	0.7578	**<0.0001**	-0.0528	0.4607	0.8106	**<0.0001**
Education (ref: primary)						
Secondary	1.9909	**<0.0001**	0.7331	**<0.0001**	1.2577	**<0.0001**
Third	3.1047	**<0.0001**	1.4389	**<0.0001**	1.6658	**<0.0001**
Married (ref: others)	0.4090	**0.0203**	0.1359	0.1181	0.2732	**0.0435**
Hypertension	1.2266	0.1449	0.4853	0.2424	0.7412	0.2514
Dyslipidemia	0.4768	0.3277	0.4033	0.0935	0.0736	0.8441
Diabetes	0.6831	0.3670	-0.1581	0.6722	0.8412	0.1481
Heart diseases	-0.8029	0.3115	-0.1036	0.7912	-0.6993	0.2511
smoking status (ref: smoke)						
former	0.2512	0.1827	-0.0056	0.9521	0.2568	0.0762
never	0.0121	0.9330	-0.0404	0.5702	0.0525	0.6354
current drinking (ref: >once per month)						
≤once per month	0.3268	0.0999	0.1861	0.0576	0.1407	0.3562
no current drinking	0.2778	**0.0323**	0.0924	0.1490	0.1854	0.0629
CES-D	-0.0997	**<0.0001**	-0.0334	**0.0000**	-0.0663	**<0.0001**

Table S4: Association of volume of physical activity intensity with cognition scores.

	Vigorous PA			Moderate PA			Mild PA		
	β	95% CI	P	β	95% CI	P	β	95% CI	P
Global cognition									
none (reference)									
1-2 days/week	0.211	-0.183-0.779	0.468	-0.059	-0.755-0.873	0.901	-0.269	-0.812-0.426	0.448
3-5 days/week	0.516	0.269-0.91	0.010	0.789	0.577-1.485	0.026	0.734	0.473-1.277	0.008
6-7 days/week	-0.119	-1.051-0.129	0.347	-0.001	-0.695-0.211	0.995	0.073	-0.198-0.334	0.583
Episodic memory									
none (reference)									
1-2 days/week	0.078	-0.109-0.349	0.570	-0.020	-0.351-0.423	0.930	-0.064	-0.323-0.266	0.704
3-5 days/week	0.163	0.046-0.351	0.087	0.341	0.241-0.672	0.043	0.101	-0.023-0.359	0.446
6-7 days/week	-0.014	-0.457-0.103	0.810	-0.063	-0.394-0.037	0.219	0.009	-0.416-0.133	0.891
Mental intactness									
none (reference)									
1-2 days/week	0.132	-0.161-0.557	0.541	-0.039	-0.558-0.656	0.912	-0.205	-0.61-0.313	0.438
3-5 days/week	0.353	0.168-0.646	0.018	0.448	0.29-0.967	0.091	0.633	0.439-1.038	0.002
6-7 days/week	-0.104	-0.8-0.08	0.268	0.062	-0.456-0.22	0.438	0.064	0.064-0.259	0.516

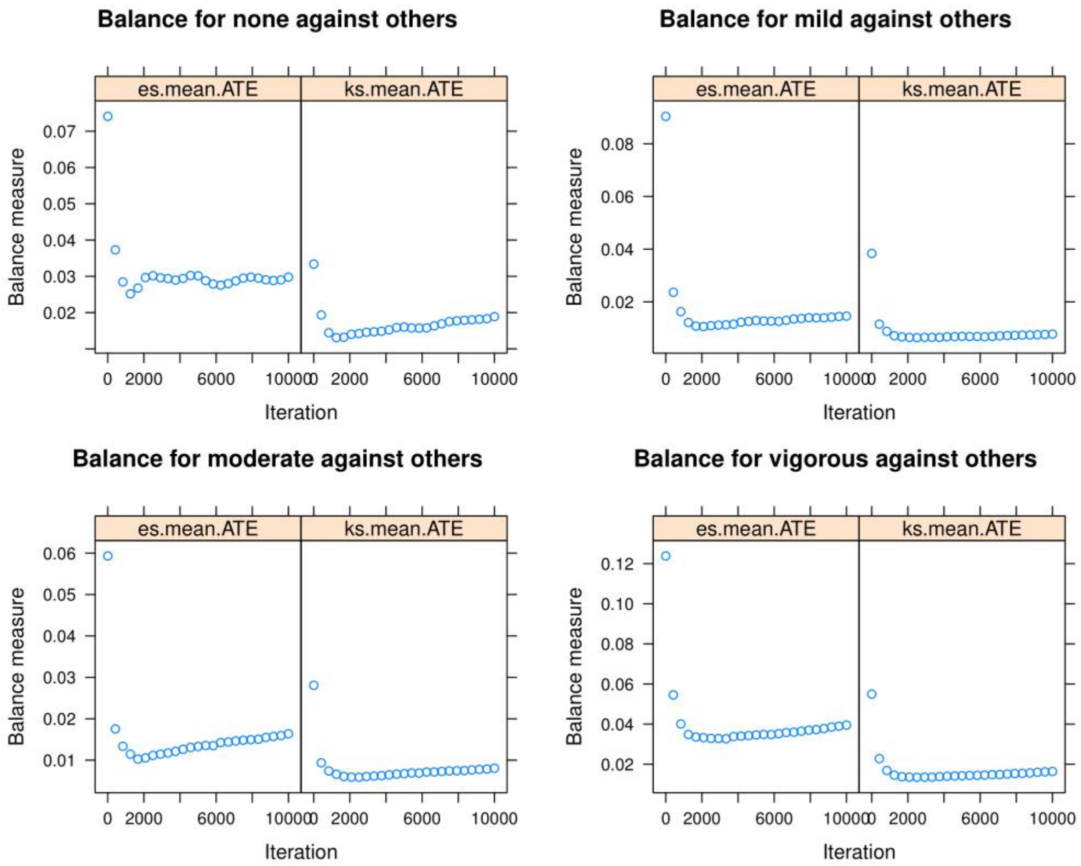

Figure S1: The balance measures of interest at different interactions.

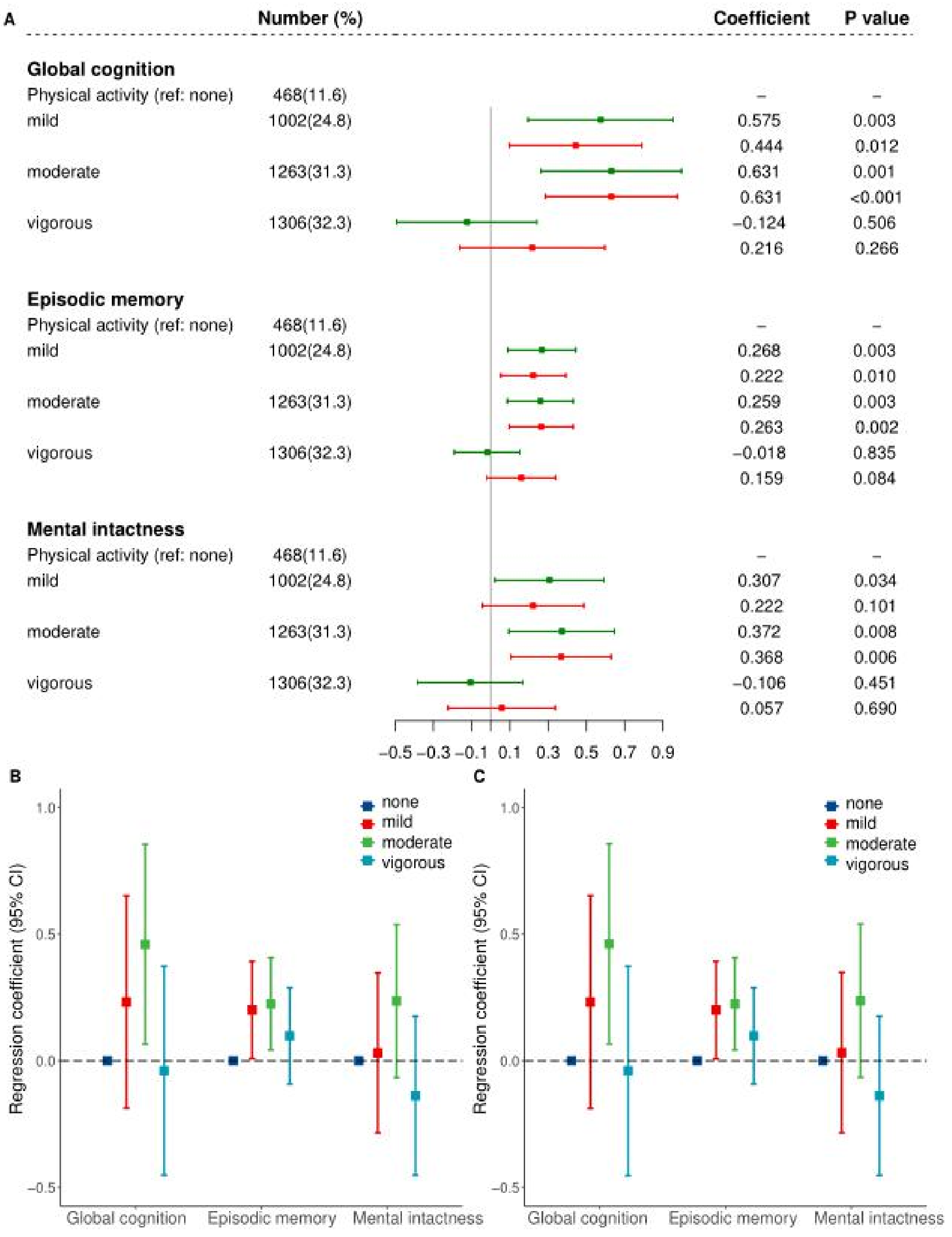

Figure 2: Results of sensitivity analyses.

A: the pooled association of PA intensity with cognitive after data imputation. the green line refers to the results in model 1; the red line refers to the results in model 2;

B, C: the association between PA intensity and cognitive after the multinomial propensity score weighting procedure using the 'es.mean' and 'ks.mean' as the stopping rule parameters, respectively.

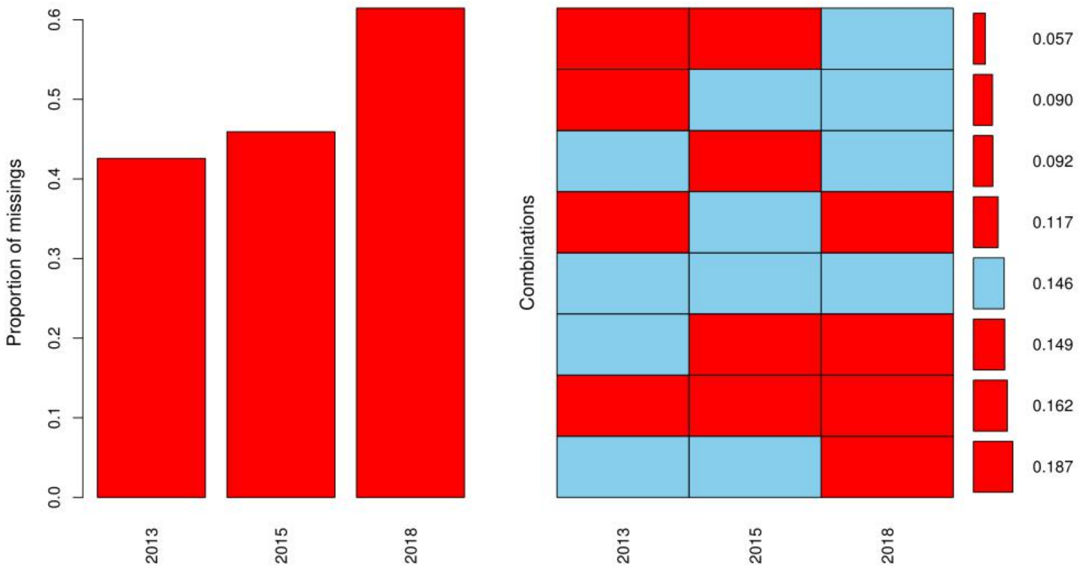

Figure S3: The missing pattern of cognition function tests during follow-up.
